# An adept approach to ascertain and elude probable social bots attacks on twitter and twitch employing machine learning approach

**DOI:** 10.1016/j.mex.2023.102430

**Published:** 2023-10-10

**Authors:** Monikka Reshmi Sethurajan, Natarajan K．

**Affiliations:** a Research Scholar, Department of Computer Science and Engineering, School of Engineering and Technology, CHRIST (Deemed to be University), Kengeri Campus, Bengaluru, Karnataka 560074, India; b Associate Professor, Department of Computer Science and Engineering, School of Engineering and Technology, CHRIST (Deemed to be University), Kengeri Campus, Bengaluru, Karnataka 560074, India

**Keywords:** Adaptive fuzzy neuro model integrated with a hist gradient boosting classifier, Harris- Hawkin's optimization with Bi-LSTM, Social bot detection, Cyber attacks, Machine learning, Classification accuracy, Fact checking, Feature extraction, Hist gradient boosting classifier

## Abstract

There has been a tremendous increase in the popularity of social media such as blogs, Instagram, twitter, online websites etc. The increasing utilization of these platforms have enabled the users to share information on a regular basis and also publicize social events. Nevertheless, most of the multimedia events are filled with social bots which raise concerns on the authenticity of the information shared in these events. With the increasing advancements of social bots, the complexity of detecting and fact-checking is also increasing. This is mainly due to the similarity between authorized users and social bots. Several researchers have introduced different models for detecting social bots and fact checking. However, these models suffer from various challenges. In most of the cases, these bots become indistinguishable from existing users and it is challenging to extract relevant attributes of the bots. In addition, it is also challenging to collect large scale data and label them for training the bot detection models. The performance of existing traditional classifiers used for bot detection processes is not satisfactory. This paper presents:

• A machine learning based adaptive fuzzy neuro model integrated with a hist gradient boosting (HGB) classifier for identifying the persisting pattern of social bots for fake news detection.
• And Harris Hawk optimization with Bi-LSTM for social bot prediction.
• Results validate the efficacy of the HGB classifier which achieves a phenomenal accuracy of 95.64 % for twitter bot and 98.98 % for twitch bot dataset.

## Graphical abstract

**Figure:**
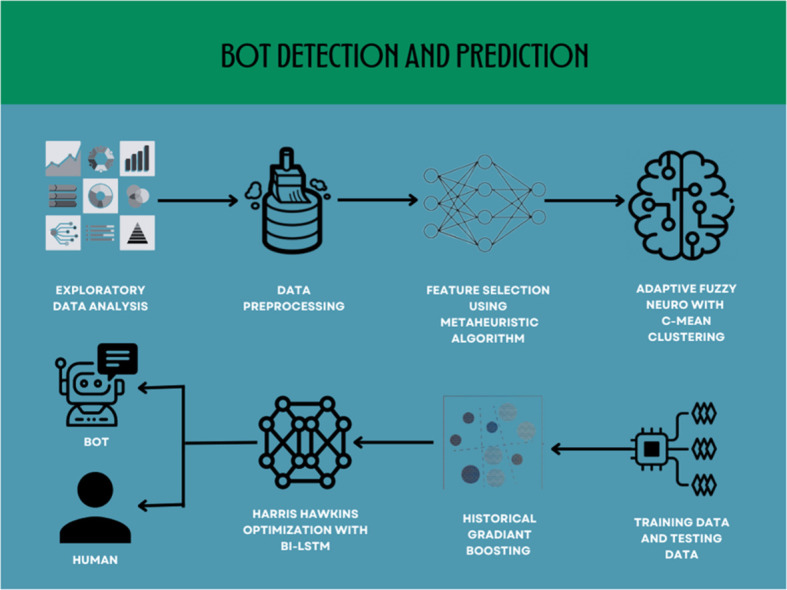

Specifications table

**Table:** 

Subject area:	Computer Science
More specific subject area:	Machine learning
Name of your method:	Adaptive fuzzy neuro model integrated with a hist gradient boosting classifier, Harris- Hawkin's optimization with Bi-LSTM
Name and reference of original method:	Neural network, Bi-LSTM, Gradient boosting
Resource availability:	The URL of the dataset is given below: https://www.kaggle.com/datasets/danieltreiman/twitter-human-bots-dataset/code https://www.kaggle.com/code/whegedusich/twitch-chat-spam-bot-detection/notebook Software used is Jupyter in Python Language.

## Introduction

The emergence of the internet and other web technologies have led to the increase in the popularity of online platforms. A variety of information can be swiftly shared through these platforms and can reach millions of people in a shorter time [1]. However, it cannot be ensured that all information shared through these platforms are genuine and factually correct [2]. Recently, social bots are used extensively in social media applications for different purposes such as spreading of false information, exploiting social media information for illegal purposes, and spreading malicious URLs and rumors to mislead the users [3]. Social bots are also exploited for increasing the popularity of the users by increasing their followers, by sharing and commenting on specific posts [4]. Furthermore, the social bots are also used to manipulate the public opinion about different aspects in sensitive aspects such as political activities. These bots help the hackers or intruders to spread misinformation through fake tweets on Twitter. This factor has a significant impact on the decision of the voters [5,6]. Several research works are dedicated for bot detection in social media platforms [7], [8], [9]. Most of these techniques are limited to the detection of social bots in prominent social media platforms such as Twitter [10,11], and Facebook [12] since these two are the major source for aggregating and circulating information. The technological advancements have introduced different techniques for verifying the authenticity of the information shared. However, it is complicated to detect bots and fact check the data accessed from the abundant social media data [13]. This research identifies three main challenges related to the detection of social bots. The first challenge is related to the extraction of bot-related attributes. Due to the similarity between the registered account holders and social bots, it is complicated to distinguish these two and extract features. In most of the cases, social bots disguise themselves as normal users to escape from being detected. Hence it is essential to incorporate an efficiency feature extraction model for detecting social bots [14]. However, existing models identify social bots from a restricted view and hence are not effective for real-time bot detection. In addition, few studies have used only fewer features for building detection models which affects the performance of these models. The second challenge is related to the collection of large-scale labeled and reliable data. Conventional approaches rely on manual labeling of data, which is a tedious task and are susceptible to manual errors [15]. Hence it is essential to train the model on a large-scale dataset. The third challenge is related to the performance efficiency of conventional detection techniques, which exhibits moderate performance.

Recently, most of the bot detection and fact checking platforms are employing artificial intelligence (AI) to cross check the originality of the shared information and both machine learning (ML) [16] and deep learning (DL) [17] models are gaining huge significance among the researchers because of their superior learning and classification abilities. ML and DL based text classification can help in distinguishing social bots from normal users and fact checking the shared information.

However, it is challenging to segregate the bots from the already existing users and this raises concern on the performance of bot detection techniques. In addition, it is also essential to determine whether the existing ML and DL models can identify the pattern of social bots. This problem motivates this research to design an efficient approach for detecting fake news from social bots. The paper presents a ML-based adaptive fuzzy neuro classifier integrated gradient boosting classifier for identifying the persisting pattern of social bots for recognizing valid information.

## Method details

**Fig. 1:**
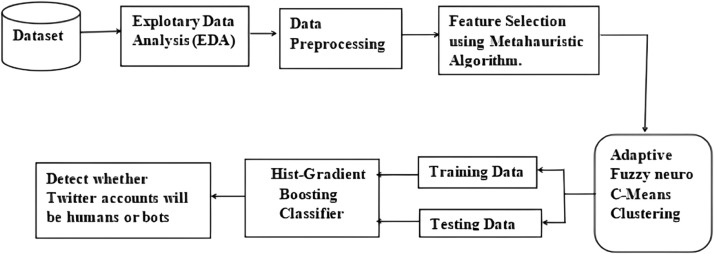
Schematic representation of the proposed approach.

**Fig. 2:**
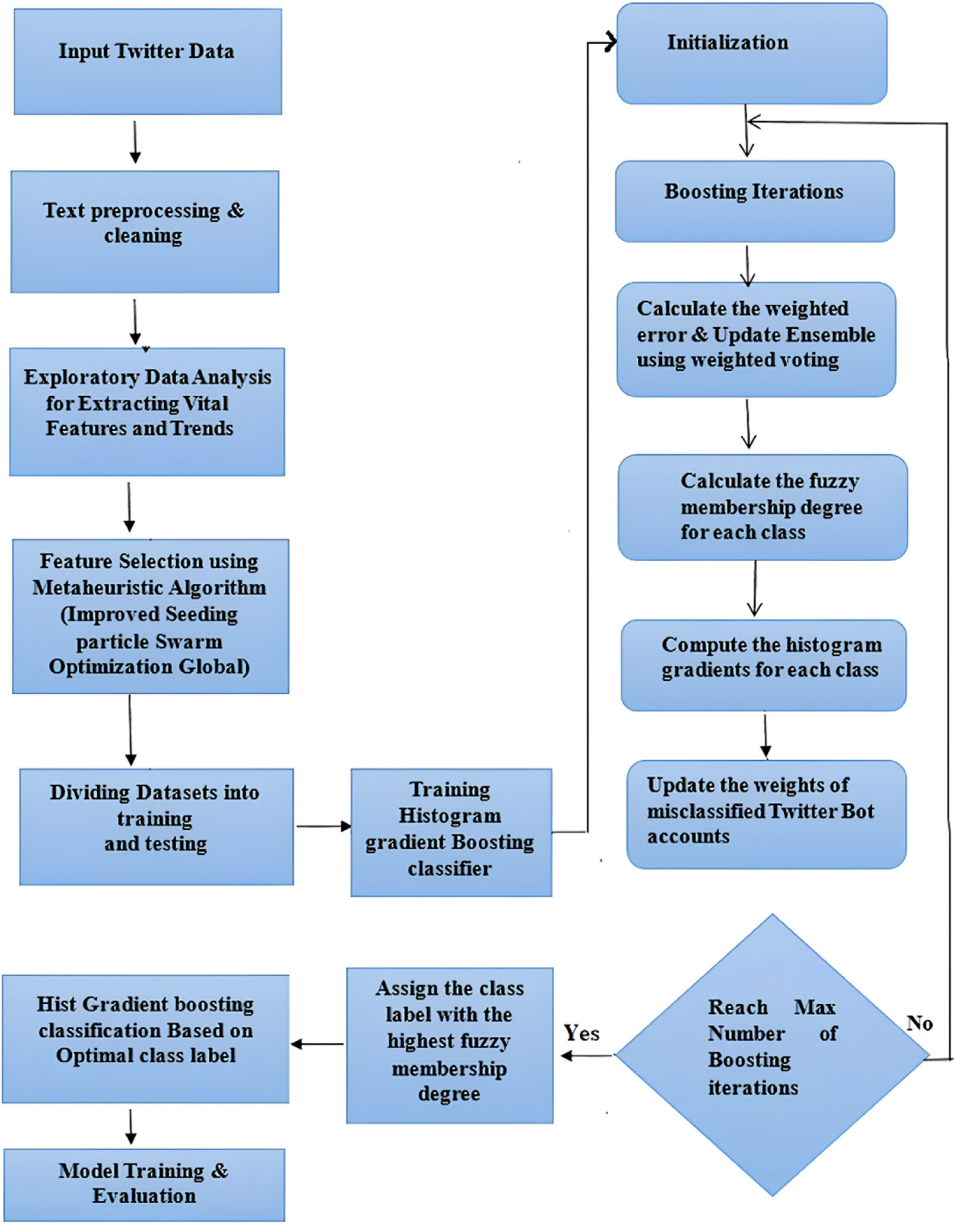
Flowchart of the proposed approach.

This research focuses on designing a feature extraction based social bot detection model which integrates an adaptive fuzzy classifier with a Hist Gradient Boosting Classifier. The proposed approach will incorporate the classification and detection attributes of the Fuzzy C means gradient boosting classifier along with an improved seeding PSO algorithm for extracting real time information from the social media. The improved PSO algorithm is based on the seeding strategy to optimize the functioning of the hybrid classifier. In this research, the improved PSO extracts the relevant features of social bots and are classified using the gradient boosting classifier. The Fuzzy technique helps the gradient boosting model to process the raw unstructured data and convert into structured format. The integrated approach helps in dealing with large amounts of information. The classified information is then used to train the detection model for detecting the social bots. The schematic representation and flow chart of the proposed framework is described in Figs. 1 and 2 respectively.

## Data collection

The data set containing the activity pattern of different users is extracted from the dynamic activity reports of large cyber environments. The data is collected from two datasets namely the Twitter Bot dataset and Twitch chat bot dataset. The URL of the dataset is given below:

https://www.kaggle.com/datasets/danieltreiman/twitter-human-bots-dataset

https://www.kaggle.com/code/whegedusich/twitch-chat-spam-bot-detection/notebook

The Inputs are twitter and twitch dataset which contains a lot of features and twitter dataset contains 37438 data samples as an input to the model and twitch chat data contains 2234025 data samples as an input to the model. Twitter bot datasets usually include information about user accounts, such as usernames, display names, follower counts, and account creation dates. The twitter data also contains the actual messages (tweets) posted by user accounts. These tweets are labeled to indicate whether they are from bot accounts or genuine users. The twitter and twitch chat bots automatically send the chats to the users. These bots are designed for performing malicious activities such as spamming, to spread fake news, to carry out false communication and illegally access the credentials. From the tweets and user profiles, features related to the frequency and timing of tweets, including the number of tweets per day or hour are extracted. Each account or tweet is labeled as either a bot or a genuine user. These labels are typically determined through manual annotation or using predefined criteria. On the other hand, Twitch chat bot datasets include information about users in a Twitch channel's chat and user data consist of usernames, display names, follower counts (if available), and account creation dates. Each chat message is labeled as originating from a chat bot or a genuine viewer. Labels are assigned based on predefined criteria or manual annotation.

### Data preprocessing

**Fig. 3:**
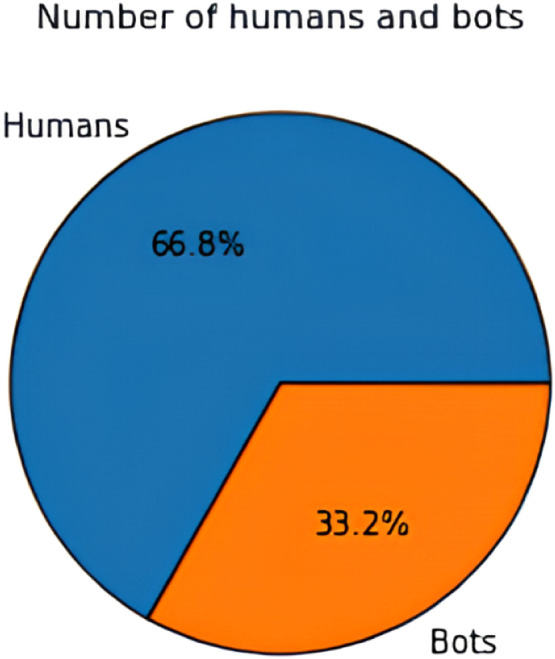
Distinguishing humans and social bots from twitter bot dataset.

**Fig. 4:**
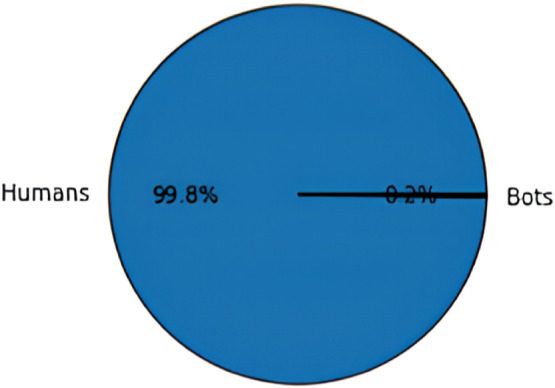
Distinguishing humans and social bots from twitch bot dataset.

**Fig. 5:**
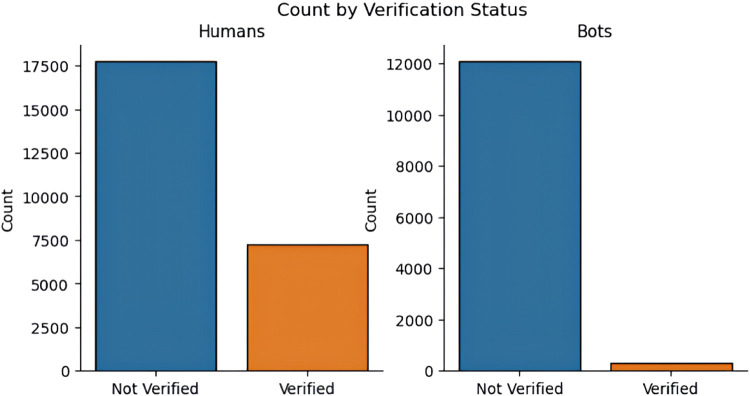
Verification count of humans and social bots.

The data acquired is subjected for processing to eliminate noise, inconsistency, and redundancy from the textual data. Preprocessing makes the data appropriate for the classification process and hence it is critical to achieve desired performance of ML algorithms in terms of classification and detection accuracy. For preprocessing, this research implements an exploratory data analysis (EDA) technique which helps in analyzing the data with the help of visual techniques [18]. In this work, the EDA is used to explore and understand the behavior of the social bots in a social media network, to identify the data patterns, trends of bot detection and to validate the assumptions made with the help of statistical tools and graphical representations. EDA also helps in identifying the missing values and null values to improve the classification accuracy. The missing values in the dataset can occur when no data is provided for one or more elements in a row or column. If the information is not available for each element, then that particular block in a row or column shows null value. While performing EDA, the missing values or the null values are skipped automatically. The graphical representation of EDA for distinguishing social bots and humans from both twitter bot dataset and twitch bot dataset are shown in below Fig. 3, Fig. 4, Fig. 5:

As shown in the above Fig. 3, Fig. 4, Fig. 5, EDA identifies 66.8 % of the data as normal users (humans) and the remaining 33.2 % as social bots from the twitter bot dataset and 99.8 % of the data as human users and 0.2 % of the data as social bots. After preprocessing, the data is categorized as dependent and independent data. Here, the independent data or variable is defined as the variable which does not change with the changes in another variable. These variables are also referred to as factors or predictions. On the other hand, the dependent variables are the ones which experience changes in its value based on the result of an action performed by the user. In addition, the data is split into training and testing data. In this work, the social bot detection model is evaluated using both the training and testing data.

### Feature extraction and feature selection

In this step, the relevant features are extracted for the classification process. This is done to reduce the dimensionality of data and improve the computational performance by eliminating unwanted features from the data [19]. The ML based classifiers analyze the data and then investigate the data to identify the interrelations between the data features to improve the classification performance. This research employs a metaheuristic based improved PSO algorithm [20] for selecting and extracting bot-related features. The application of metaheuristic methods for feature selection maximizes the computational efficiency, avoids the requirement of additional resources for feature selection and minimizes execution time and training time. In addition, feature extraction also improves the classification and detection accuracy by eliminating the redundant information from the dataset. Metaheuristic algorithms are capable of achieving high classification accuracy [21] and the accurate classification of social bots will help in fact checking the information. The improved PSO in this approach aids the ML classifiers to achieve better computation speed since it is performed in parallel to minimize the complexity of the detection model.

### Improved PSO with seeding strategy

A PSO algorithm is a metaheuristic approach which is used to optimize various single-objective problems. It is an evolutionary optimization technique which imitates the nature of swarm of particles which return to their designated regions in a stochastic manner. In PSO, the velocity and position of the particles are continuously updated and during every iteration, every individual particle will be accelerated towards the previous best and global best position of the particle. After every iteration, the values are updated with the new values as shown in Eq. 1. The updated value is substituted in the previous iteration to obtain the next position. The process is continued till the process reaches an optimal solution by achieving minimum error.

(1) $$v_{ij}=\omega *v_{ij}+c_{1}*r_{1}\left(p_{ij}-x_{ij}\right)+c_{2}*r_{2}\left(p_{g}-x_{ij}\right).$$

(2) $$x_{ij}=x_{ij}+v_{ij}$$

where $v_{ij}$ is the velocity and $x_{ij}$ is the position, rc_1_ and c_2_ are defined as the constants, ω is defined as the inertia, r_1_ and r_2_ are the random variables which ranges from 0 and 1. P_g_ and p_ij_ defines the global best and current position of the particle, x_ij_ is the current position and v_ij_ defines the velocity of the particles.

The improved PSO utilizes a set of optimal solutions for a specific problem. The algorithm traverses the search space related to the problem and fine-tunes the parameters for maximizing the objective function (OF) of the problem. The algorithm minimizes the impact of problem by optimizing the OF. In addition, PSO exhibits a lower convergence rate and rarely falls into the local optimum. As a result, the improved PSO technique can effectively solve optimization problems. The improved PSO with seeding strategy helps in maintaining the stability of the algorithm and increases the chances of achieving global convergence using a fewer number of iterations [22].

**Fig. 6:**
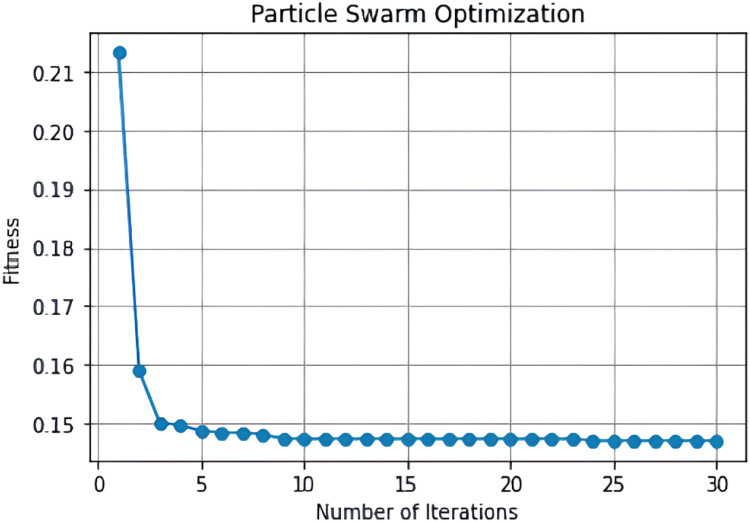
Convergence of improved PSO with seeding strategy.

After selecting and extracting the features using improved PSO, the features were reduced from 35 to 10 relevant features to maximize the detection accuracy. Further, the features are split into independent and target variables and the dataset is fit to obtain the best feature set for classification. After fitting the best feature set into the model, the improved PSO estimates the best features based on the convergence between number of iterations and fitness value. The features with best fitness values are selected and are represented using a data frame. After creating the data frame, the data is split using a standard scaler to calibrate the range of all data values into a standard form. Here, the data is split into a ratio of 70:30 for training and testing the performance of the HGB classifier. Appropriate splitting of data accelerates the performance of the model to identify the best features accurately, which can be defined by plotting the convergence is shown in Fig. 6.

The improvised PSO was tested for 30 iterations and the algorithm achieves convergence after initialization. The best values of PSO were obtained for 28, 29 and 30^th^ iterations with a value of 0.147 and feature size 11. The accuracy of improved PSO in selecting best features was found to be 85.48 % and the selected best features are given as input to the classifier for classifying social bots.

The process involved in the selection of best features by the improved PSO are discussed below:

Step 1: Determining the Number of Features

In this step, the frequency of selected features within a limited zone are identified. The parameters that influence the selection of features are identified, which are denoted as ‘r’ and ‘v’. Parameter ‘r’ can be adjusted based on the total number of samples in the dataset, ‘v’ determines the percentage of features favored by the user in the final output. The primary goal is to obtain the most relevant features for the final output.

Step 2: Population initialization

In this step, the population is initialized which majorly impacts the optimization process. Initial solutions, generated based on some criteria, help the population move towards optimal solutions. A Chaos theory is used to enhance the uniform distribution of the population. Chaos-based initialization enhances the likelihood of convergence.

Step 3: Updating the particle positions (PSO)

The PSO is a key component of this approach wherein the particles represent solutions in the search space and the particles move based on their own experience and the position of neighboring particles. Equations govern the velocity and position updates for each particle.

Step 4: Global search seeding

This step involves evaluating the relationship between features to identify informative features. Salient features are saved in a storage list based on their frequency. Features are selected from the storage list using a Boltzmann distribution and added to the new generation. This step aims to improve space search and maintain diversity in the population.

Step 5: Gbest mutation

Gbest (global best) and Gworst (global worst) particles are considered. Mutation or reversing Gbest is utilized to explore uncharted regions. Mutation introduces diversity and helps identify suboptimal positions.

Step 6: Fitness value (FV)

The FV evaluates the robustness of each solution. Feature values are normalized between -1 and 1 before evaluation. A 10-fold cross-validation is employed for estimating the fitness value of each solution.

Correspondingly, the mathematical model for Improved seeding PSO is described as follows:

Step 1: Determining the number of features

Let, N be the total number of features in the dataset, ‘*r’* be the adjustable parameter for selecting features, *‘v’* be the adjustable parameter representing the percentage of features favored by the user and CR be the number of data samples. The mathematical representation for selecting features is as follows:

(3) $$Selected\;Features=\left\{f\_i|frequency\;\left(f_{i}\right)\rangle r*CR\right\}$$

where f_i is a feature in the dataset

Step 2: Population Initialization

Population initialization involves generating initial solutions. In this step, chaos theory is used to create random but evenly distributed initial solutions.

Step 3: Updating the Particle Positions (PSO)

PSO involves updating particle positions and velocities. The velocity update equation for each dimension i of a particle j is as follows:

(4) $$v_{ij}\left(t+1\right)=v_{ij}\left(t\right)+C_{1}*r_{1}*\left(P_{best\;ij}-x_{ij}\left(t\right)\right)+C_{2}*r_{2}*\left(G_{best\;ij}-x_{ij}\left(t\right)\right)$$

Where: v_ij_ (t+1) is the updated particle velocity for j in dimension i at time t+1, c_1_ and c_2_ are acceleration constants, r_1_ and r_2_ are random numbers, P_best ij_ is the personal best position of particle j in dimension i, G_best_i_ is the global best position in dimension i, and x_ij_(t) is the current position of particle j in dimension i at time t.

### Step 4: Global search seeding

This step involves identifying informative features and storing them in a storage list based on their frequency. It uses the Boltzmann distribution for feature selection. In this step, the goal is to identify the most distinct and informative features and store them in a storage list. The Boltzmann distribution is used to select features based on their frequency. The Boltzmann distribution formula for feature selection is as follows:

(5) $$P\left(f_{i}\right)=\frac{e^{\alpha *F_{i}}}{\sum_{j=1}^{Nf}e^{\alpha *F_{i}}}.$$

where, P(f_i_) is the probability of selecting feature f_i_ based on its frequency, N_f_ is the total number of features, F_i_ is the frequency of feature fi, α is a control parameter (typically related to temperature). Features with higher probabilities are selected and moved to the new generation.

Step 5: Gbest mutation

Mutation or reversing Gbest is used to explore uncharted regions. Gbest mutation involves making small random changes to the Gbest particle or reversing its direction to explore uncharted regions. Let: Gbest be the current global best solution, Gworst be the worst fit particle. The mathematical representation for Gbest mutation can be a probabilistic operation:

(6) $$If\;rand<s_{v}then\;x_{mutated}=1,\;else\;it\;is\;0$$

where, rand is a random number, s_v- is a mutation probability, x_mutated- It is the mutated feature.

Step 6: Fitness value

The FV evaluates the standard of each solution and it measures the classification accuracy of selected features and is given by Eq. 7.

(7) $$Fitness\left(X\right)=\frac{1}{N}\sum_{i=1}^{N}\delta \left(X_{i},\;Y_{i}\right).$$

N is the number of data points, X_i_ is the i^th^ data point, Y_i_ is the true class label of X_i_, δ(X_i_, Y_i_) is the Kronecker delta function, which returns 1 if the predicted class matches the true class and 0 otherwise. The flowchart of the IS-PSO algorithm is illustrated as follows:

## Classification of social bots using an adaptive fuzzy neuro with hist gradient boosting classifier

Based on the extracted features, this research employs an adaptive fuzzy neuro classifier integrated with a Hist Gradient Boosting classifier. The gradient boosting algorithm is an ensemble of decision trees which are used to assign and predict a target label [23]. The gradient boosting algorithm is selected because of its capacity to handle massive loads of data to make predictions with high accuracy.

In the proposed hist gradient boosting algorithm, a histogram is used for determining the frequency of data occurrences over discrete periods called bins, wherein each bin represents the frequency of the image pixel value. By minimizing the number of features, the speed of the algorithm is increased and based on this concept, multiple decision trees are aggregated with histograms for a hist gradient boosting (HGB) classifier. The decision trees in the hist gradient boosting algorithm are trained to classify the social bots into normal users or bots. The adaptive fuzzy classifier assists the gradient boosted decision trees to achieve high speed and better performance. The fuzzy classification algorithm is used in this research, to minimize the consistency of training and testing the model. Here, the data is split into two types namely a center value and a label value. The center value is the input value and the label value is the target value, which is split using the adaptive fuzzy classifier. The input and target value are classified before training the model. The fuzzy classifier uses a clustering method to classify the data based on their label value. In fuzzy c means (FCM) classifier, the membership degree for each data sample is determined and the cluster to which the data belongs to is calculated using the FCM membership degree. The degree of FCM membership and the cluster matching can be minimized as shown in Eqs. 3a and 4a.

(3a) $$V_{i}=\frac{\sum_{k=1}^{n}u^{m}ik\;x_{k}}{\;\sum_{k=1}^{n}u^{m}ik};1\leq i\leq c$$

(4a) $$u_{ik}=\frac{1}{\sum_{j=1}^{c}{\left(\frac{d_{ik}^{2}A}{d_{jk}^{2}A}\right)}^{\frac{2}{m-1}}}$$

The main objective of dividing the dataset into clusters to achieve an optimal loss function and make this function reach the minimum value.

(5a) $$J\left(U,\;V\right)=\sum_{j=1}^{N}\sum_{i=1}^{c}{\left(u_{ij}\right)}^{2}{\left(d_{ij}\right)}^{2}$$

Here, u_ij_ denotes the degree of membership of j.

To enhance the classification accuracy of the adaptive fuzzy classifier, multiple individual decision trees (DT) are fused into a single model. For each individual tree, the value of the loss function defined in Eq. 5a is reduced. All trees are added in the classifier to increase the classification accuracy. In gradient boosting algorithms, the weights of the data samples play an important role in enhancing the performance. Here, the weights are assigned to all independent features or variables which are given as input to the DT for predicting the outcome. The weight of the variables predicted by the DT is increased and these variables are then fed to the second decision tree. In this way, the individual classifiers/predictors are ensembled to obtain a robust and more precise model for classification. The *gradient boosting* algorithm aggregates the output of each individual DT using gradient boosted trees. After aggregating the output of each tree, the classifier will assign a unique label to each feature set based on which the data is classified into normal user or social bots.

Based on the extracted features, this research employs an adaptive fuzzy neuro classifier integrated with a Hist Gradient Boosting (HGB) classifier. The HGB classifier combines multiple DTs which are used to assign and predict a target label [23]. The HGB classifier can process large data volume to predict the target accurately. In the proposed Hist gradient boosting algorithm, a histogram is used for determining the frequency of data occurrences over discrete periods called bins, wherein each bin represents the frequency of the image pixel value. By minimizing the number of features, the speed of the algorithm is increased and based on this concept, multiple decision trees are aggregated with histograms to for a HGB classifier. The decision trees in the Hist gradient boosting algorithm are trained to classify the social bots into normal users or bots. The adaptive fuzzy classifier assists the HGB model to achieve better performance. The fuzzy classification algorithm is used in this research, to minimize the consistency of training and testing the model. Here, the data is split into two types namely a center value and a label value. The center value is the input value and the label value is the target value, which is split using the adaptive fuzzy classifier. The input and target value is classified before training the model. The fuzzy classifier uses a clustering method to classify the data based on their label value. In fuzzy c means (FCM) classifier, the membership degree for each data sample is determined and the cluster to which the data belongs to is calculated using the FCM membership degree. The degree of FCM membership and the cluster matching can be minimized as shown in Eq. 8 and 9.

(8) $$V_{i}=\frac{\sum_{k=1}^{n}u^{m}ik\;x_{k}}{\;\sum_{k=1}^{n}u^{m}ik};1\leq i\leq c$$

(9) $$u_{ik}=\frac{1}{\sum_{j=1}^{c}{\left(\frac{d_{ik}^{2}A}{d_{jk}^{2}A}\right)}^{\frac{2}{m-1}}}$$

The main objective of dividing the dataset into clusters to achieve an optimal loss function and make this function reach the minimum value.

(10) $$J\left(U,\;V\right)=\sum_{j=1}^{N}\sum_{i=1}^{c}{\left(u_{ij}\right)}^{2}{\left(d_{ij}\right)}^{2}$$

Here, u_ij_ denotes the degree of membership of j.

To enhance the classification accuracy of the adaptive fuzzy classifier, multiple individual decision trees (DT) are fused into a single model. For each individual tree, the value of the loss function defined in Eq. 5 is reduced. All trees are added in the classifier to increase the classification accuracy. In gradient boosting algorithms, the weights of the data samples are the critical components that can affect the classification process. Based on the weights the features or variables are selected as input for the DT for predicting the outcome. The variables which possess highest weight are given to the DT and the predictions made by each DT is combined to generate finalized output. The gradient boosting algorithm aggregates the output of each individual DT using gradient boosted trees. After aggregating the output of each tree, the classifier will assign a unique label to each feature set based on which the data is classified into normal user or social bots.

The pseudocode for Fuzzy Adaptive Histogram Gradient Boosting Classifier for Twitter Bot Detection is given below:

**Table:** 

**Pseudocode:**
Begin:
Text Preprocessing
Extract relevant features from Twitter accounts using Metaheuristic algorithm
To perform feature scaling or normalization
Initialize the ensemble of weak classifiers (Hist Gradient Boosting Classifier)
Set the number of boosting iterations (T)
For t = 1 to T:
Calculate the weighted error of the ensemble based on fuzzy memberships
Select the best weak classifier that minimizes the weighted error
Update the ensemble with the selected classifier using weighted
Voting
For each Twitter account in the training set:
Calculate the fuzzy membership degree for each class label (bot or non-bot) based on the ensemble's predictions.
Compute the histogram gradients for each class based on the fuzzy membership degrees.
While (Not reaching a maximum number of boosting iterations):
Update the weights of misclassified Twitter accounts based on the histogram gradients
Repeat
While (Termination Condition is Reached):
Assign the class label with the highest fuzzy membership degree for achieving desired accuracy.
Prediction
To Apply Hist-Gradient Boosting classifier Based on Optimal class label
Model Evaluation using test datasets
Performance metrics of Model
End

### Social bot prediction using adaptive HHO-RNN with bidirectional LSTM

This work implements a Harris hawk's optimization (HHO) with a Bi-LSTM model for accurately predicting the social bots from the data.

Harris hawk's optimizer (HHO) Algorithm

**Fig. 7:**
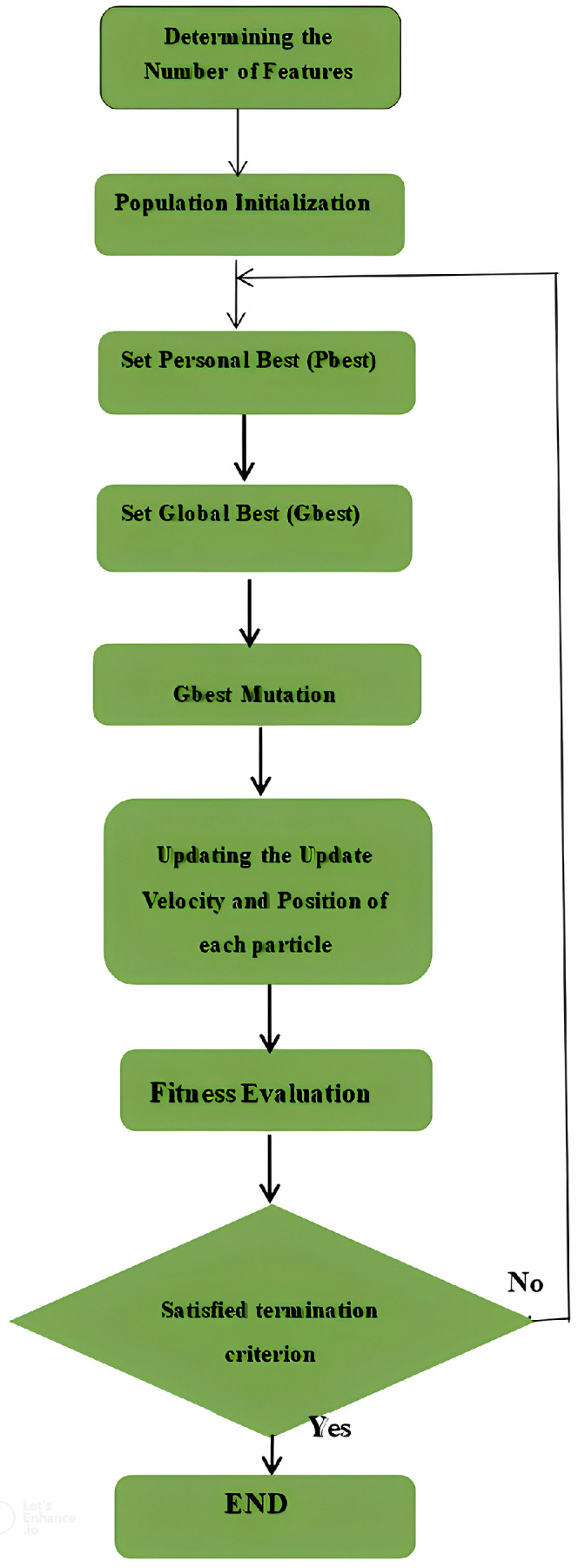
Flowchart of the IS-PSO algorithm.

The HHO was developed by [24]. The principle of the HHO algorithm is to imitate the behavior of Hawk's group with respect to its hunting behavior and the phenomenon of diverting from the prey to explore the solutions for the specific problem [25]. In this algorithm, the chasing action of a hawk is considered as a search agent and the prey represents the best position. The HHO algorithm is extensively used in various optimization tasks such as pattern recognition, feature selection, segmentation tasks, and prediction tasks. In addition, the HHO algorithm can solve both continuous and discrete problems [26]. The HHO algorithm exhibits superior performance in terms of extracting optimal features, achieving high accuracy in prediction tasks [27], [28], [29]. In addition, HHO helps in solving complex nonlinear problems and achieves better convergence while generating optimal solutions. The process involved in the HHO algorithm and the exploratory and exploitative mechanism is influenced by the behavior of Harris hawks in terms of identifying the prey, pouncing, and attacking technique (Fig. 7).

**Fig. 8:**
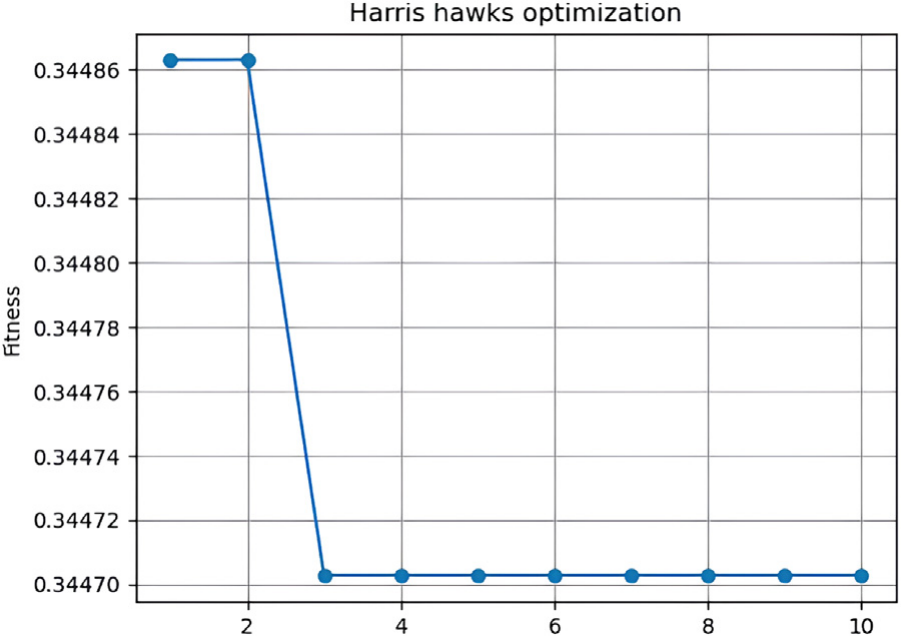
Performance of the HHO algorithm.

In this research, the HHO algorithm selects an optimal feature subset from the dataset. The execution of the HHO algorithm in terms of selecting optimal features is shown in Fig. 8.

The algorithm is executed for 10 iterations, wherein the algorithm achieves an accuracy of 66.2 % in terms of selecting the best HHO value (0.344). After feature selection, the total number of features are reduced to 3 features, which significantly improved the classification accuracy. The best features selected by the HHO are given to the Bi-LSTM for predicting the social bots.

**Fig. 9:**
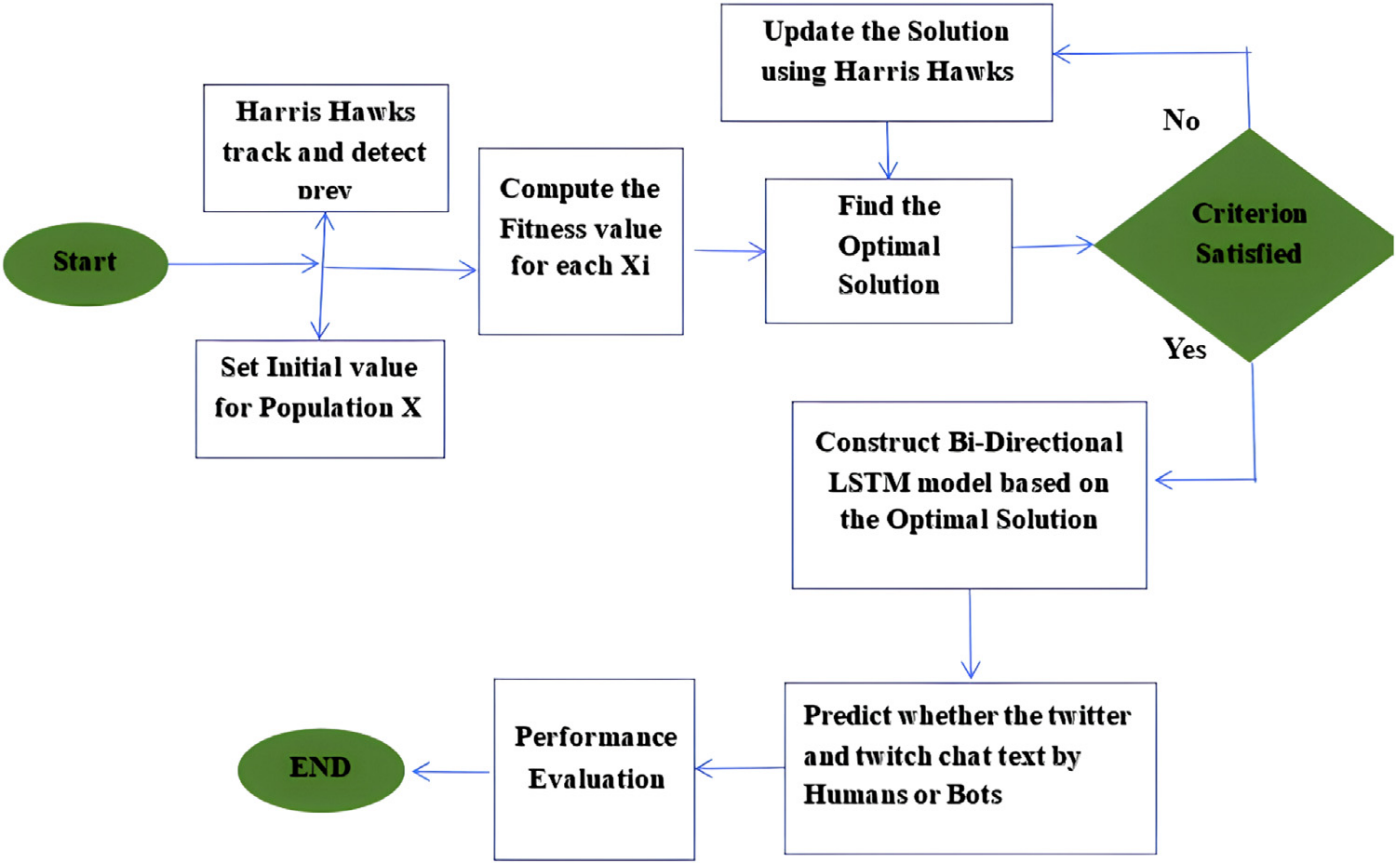
Flowchart of the integration of HHO-Bi-LSTM model.

The Bi-LSTM algorithm is selected because of its ability to learn long-time sequential data more effectively compared to LSTM and because of its potential to evaluate the long word sequences. Also, the architecture of Bi-LSTM (where the LSTM operates in forward and backward direction) is more effective since it distributes the workload equally among the LSTM layers and hence reduces the computational burden. The four-layered architecture of the proposed Bi-LSTM consists of memory units composed of three gates such as the input gate, the output gate and the forget gate. The gates control the memory of the Bi-LSTM and in turn controls the flow of information through a particular gate. This assists the Bi-LSTM to store only required information and discard others. This mechanism will overcome the problem of vanishing gradient and the data can be stored for a longer period and make it suitable for solving complex calculations. The integration of HHO and BiLSTM is shown in Fig. 9.

The steps involved in the proposed approach are discussed the following steps:

1. Feature Selection: The HHO selects the most relevant features from the available feature set. These features are chosen based on their importance for bot prediction.
2. Data Preparation: The labeled chat message or user account data is prepared, where each data point is associated with the selected features.
3. BiLSTM Training: The BiLSTM is trained on the prepared data and the model takes the selected features as input and predicts whether a chat message or user account is a bot or not.
4. Evaluation: The performance of the integrated model is determined using appropriate metrics using testing and training data.

The integration of HHO for feature selection and a BiLSTM for prediction leverages the optimization power of HHO to identify the most informative features for bot detection and the sequence modeling capabilities of BiLSTM to capture context and dependencies in chat messages. This combined approach can lead to effective and accurate bot detection systems. After training the Bi-LSTM, the potentiality is computed based on test data. If the predicted value is greater than 0.5 (*P* > 0.5) then the model predicts output as humans. On the other hand, if the predicted value is less than 0.5 (*P* < 0.5) the label is considered as a social bot.

## Method validation

The experimental analysis is conducted to validate the performance efficacy of the proposed adaptive fuzzy classifier with a hist gradient boosting classifier. The data for the experimental analysis is collected from two datasets namely Twitter bot dataset and Twitch bot dataset. The dataset consists of both normal and malicious social bot data. The input data is split into two types namely training data and testing data. Training data is used for training the ML model for detecting social bots and the performance of the ML model is validated using the testing data. Here in this research, 80 % data is used as training data and 20 % of the data is used for the testing process.

### Performance evaluation

The proposed classifier is evaluated using performance metrics denoted in Eqs. 11–14. The metrics used in the performance evaluation are computed as shown in below equations:

(11) $$\text{Accuracy}\;=\;\frac{TP\;+\;TN}{TP\;+TN+FP+FN}\ldots .$$

(12) $$\text{Recall}\;=\;\frac{\mathrm{TP}}{\mathrm{TP}\;+\;\mathrm{FN}}\ldots ..$$

(13) $$F1\;\text{score}\;=\;\frac{2\;*\;\mathrm{Precision}\;*\;\mathrm{Recall}}{\mathrm{Precision}\;+\;\mathrm{Recall}}\ldots .$$

(14) $$\text{Precision}\;=\;\frac{\mathrm{TP}}{\mathrm{TP}\;+\;\mathrm{FP}}\ldots .$$

Where, TP, TN, FP, and FN represent True positives, True negatives, False positives, and False negatives respectively. Obtained results are compared with other existing models such as DT, LR, SGD, RF, Adaboost classifier, and feed forward neural network (FFNN) classifier.

Simulation is performed for both datasets and the results of the same are discussed in below subsections:

Simulation results for Twitter bot dataset

**Fig. 10:**
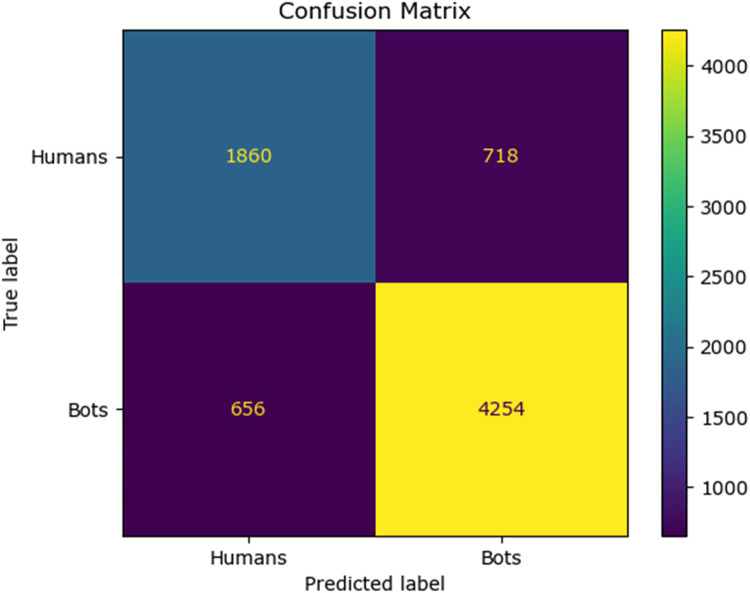
Confusion matrix of the decision tree classifier.

**Fig. 11:**
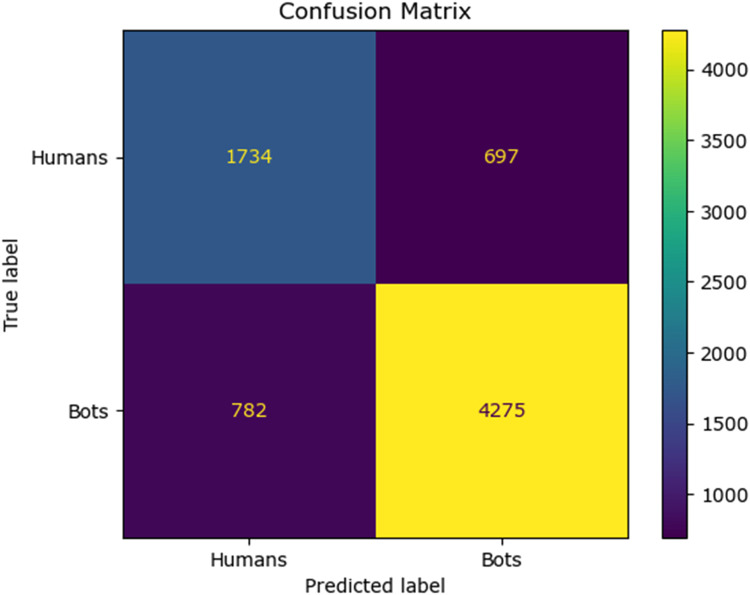
Confusion matrix of the SGD classsifier.

**Fig. 12:**
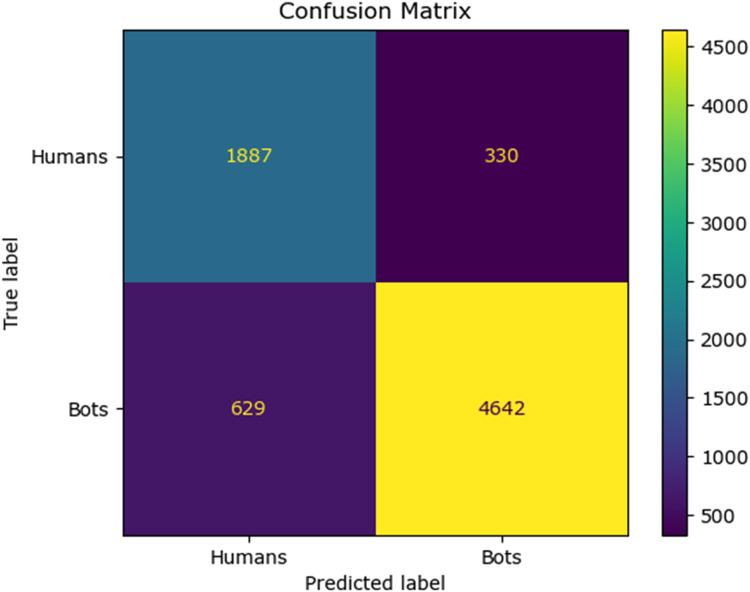
Confusion matrix of the random forest classifier.

**Fig. 13:**
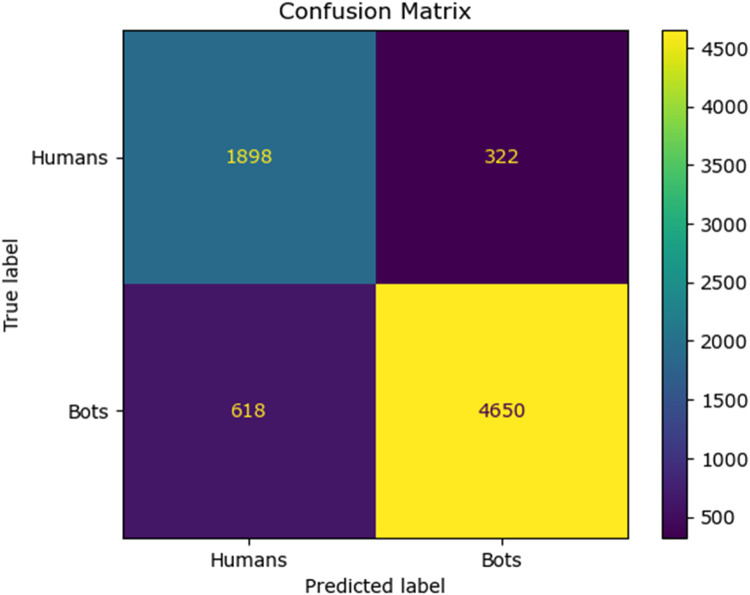
Confusion matrix of the logistic regression classifier.

**Fig. 14:**
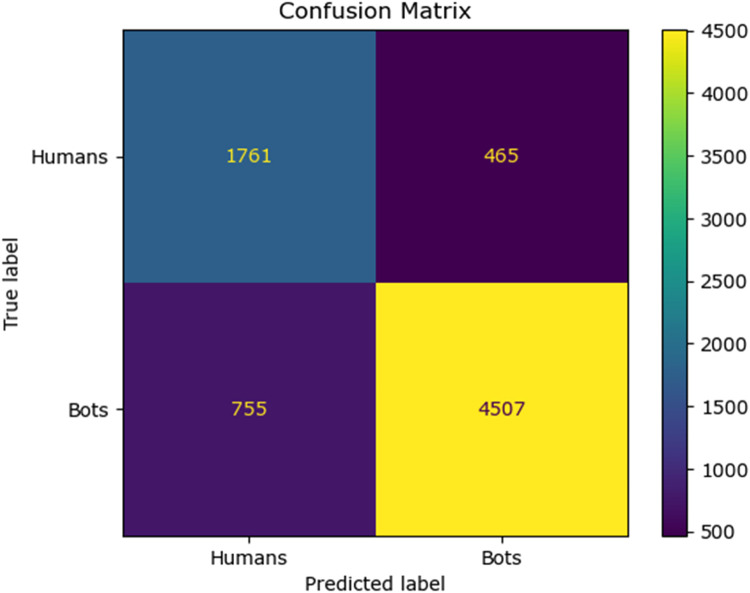
Confusion matrix of the Adaboost classifier.

The confusion matrix for the existing classifier and the proposed classifier are given in Fig. 10, Fig. 11, Fig. 12, Fig. 13, Fig. 14:

**Fig. 15:**
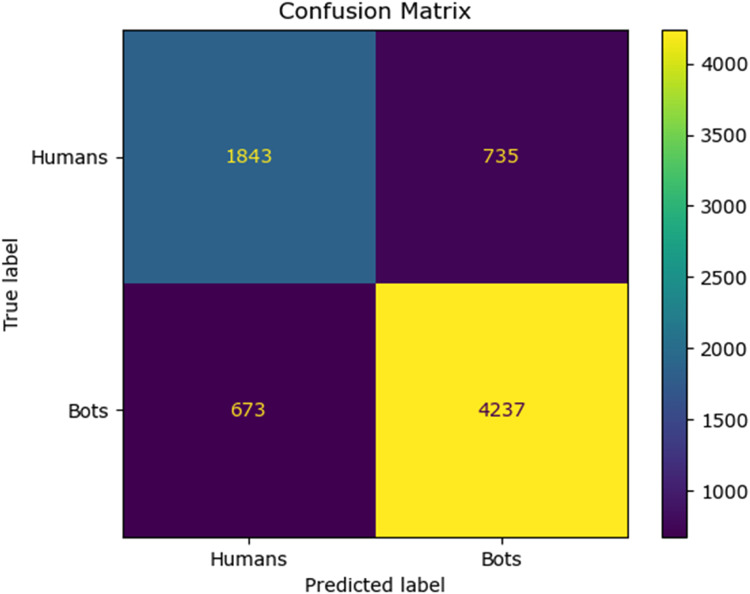
Confusion matrix of the feed forward neural network.

The values of different performance metrics obtained from simulation for the proposed classifier for twitter bot dataset are tabulated in Table 1 (Fig. 15).

**Table 1:** Performance metrics for the twitter bot dataset.

ML Models	Accuracy	Precision	Recall	f1 -score	Support
Decision Tree	81.65 %	85.55 %	86.63 %	86.09 %	7488
SGD	80.24 %	85.98	84.53 %	85.25 %	7488
Random Forest	88.01 %	93.92 %	88.76 %	88.0 %	11232
Logistic Regression	87.44 %	93.52 %	88.26 %	90.8 %	7488
AdaBoost Classifier	83.7 %	90.64%	85.65 %	88.07 %	7488
FFNN Classifier	81.19 %	85.21 %	86.29 %	85.75 %	7488
Proposed Fuzzy Neuro with Hist Gradient Boosting Classifier	97.00 %	97.00 %	97.00 %	97.00 %	37438

**Fig. 16:**
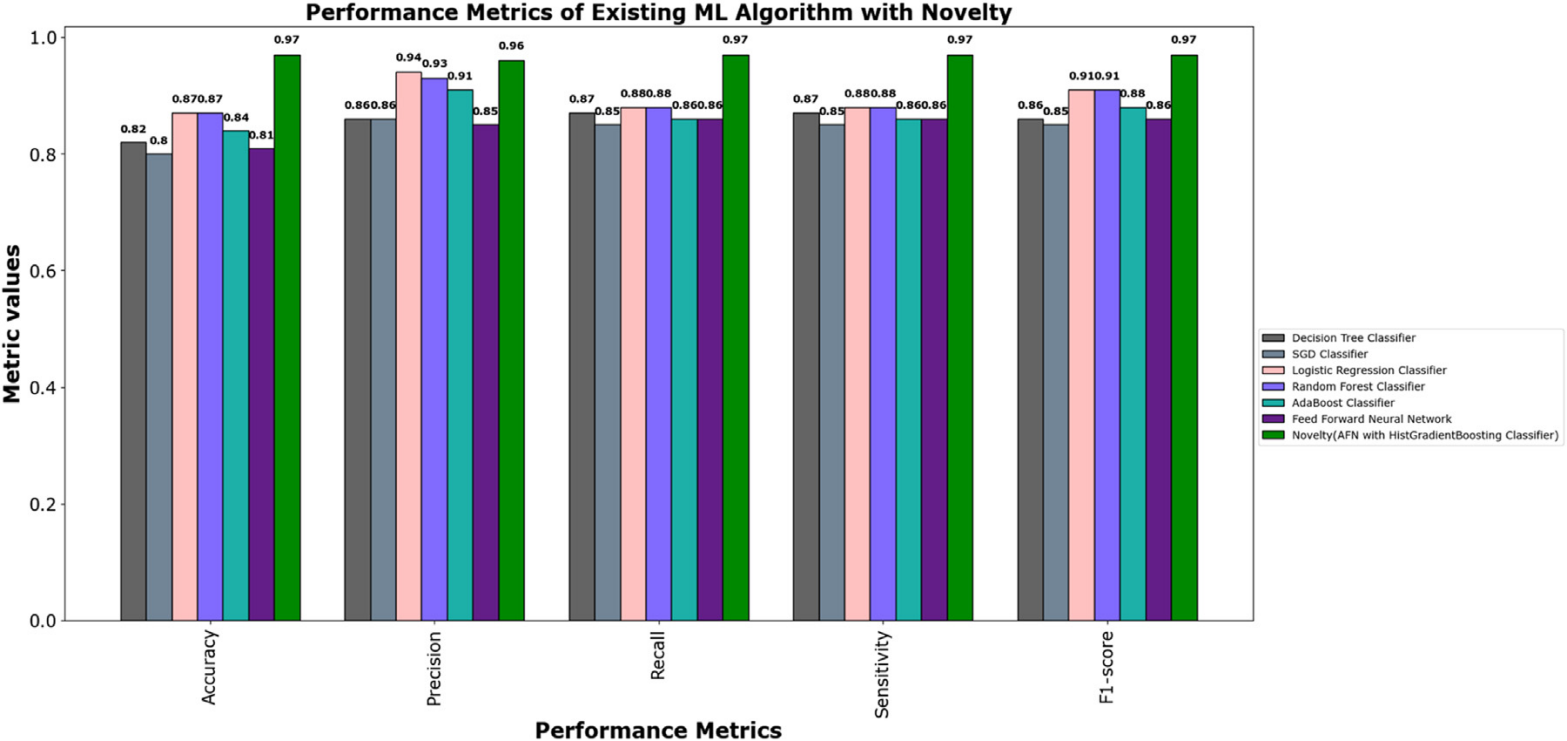
Performance evaluation of different ML classifiers for twitter bot dataset.

It can be inferred from the Fig. 16 and Table 1 that the simulation results reveal that the HGB classifier exhibited an optimal accuracy of 97.00 %, while the precision, recall, f1 score and support was found to be 97.36 %, 96.67 %, 97.01 % and 11232 respectively. The results of the HGB classifier is compared with other existing classification models for validation. Results validate the performance of the adaptive fuzzy with HGB classifier in terms of achieving a phenomenal accuracy with a very minimum false alarm rate (FAR) of 0.0181.

The performance of the HHO optimized Bi-LSTM model is analyzed with respect to training and validation accuracy, training and validation loss, and prediction accuracy.

**Fig. 17:**
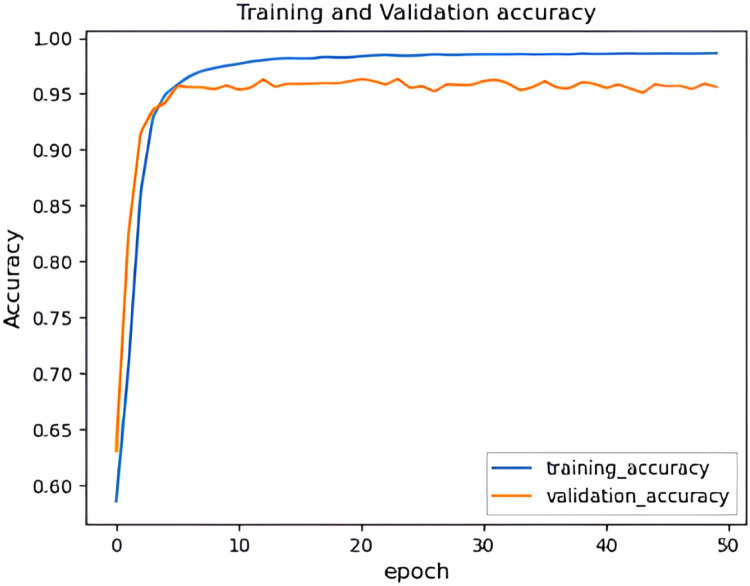
Training and validation accuracy of HHO-BiLSTM model for twitter bot dataset.

**Fig. 18:**
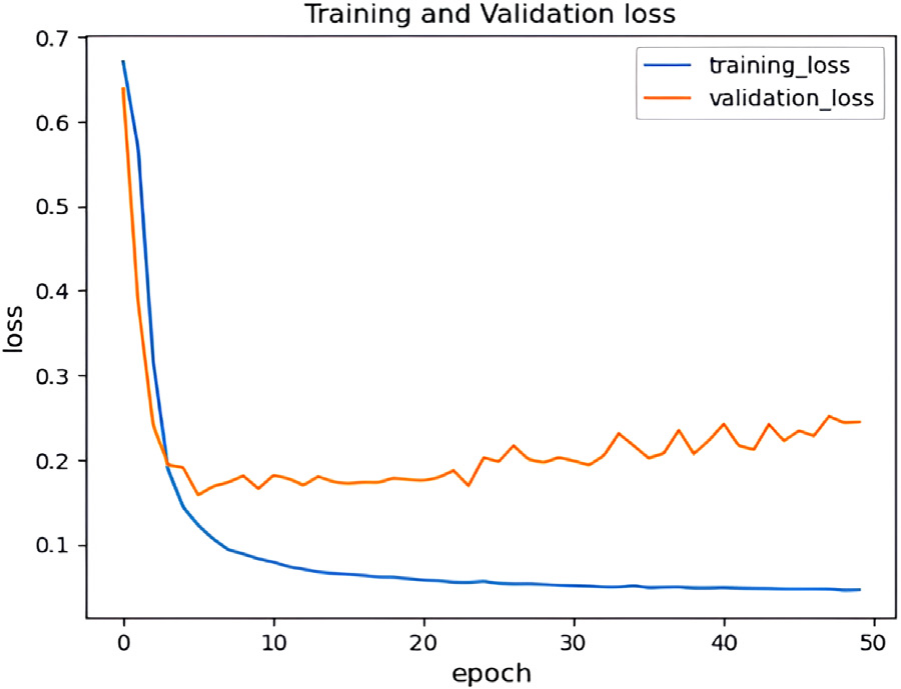
Training and validation loss of HHO-BiLSTM model for twitter bot dataset.

It can be observed from Figs. 17 and 18 that the accuracy of the HHO-BiLSTM model is based on the epochs. Here the epochs level is 50 and based on that the training accuracy is determined and is denoted using a blue line. Similarly, the red line shows the validation accuracy which is lower than training accuracy. Correspondingly Fig. 17 shows the amount of data loss (training and validation loss) based on the epochs. The training loss is denoted using a blue line and the red line represents the validated loss. As observed from the figure, the training loss is lesser than the validation loss. The graphs illustrated in above figures determine the accuracy of the proposed HHO-BiLSTM model for the dataset considered for experimental evaluation based on the epochs. The graph shows that the validated accuracy is lower than the training accuracy. The validation loss in , shows how much data is lost based on the epochs.

In addition to the performance evaluation, the results of the HHO optimized Bi-LSTM model is compared with existing classifiers such as CNN classifier, CNN with LSTM classifier, CNN with GRU and the proposed novel classifier and the results are tabulated in Table 2.

**Table 2:** Performance metrics for the prediction model for twitter bot dataset.

ML Models	Accuracy	Precision	Recall	f1 -score	Support
CNN classifier	50.49 %	50.49 %	50.57 %	50.49 %	39160
CNN with LSTM	59.81 %	59.81 %	60.90 %	59.81 %	39160
CNN + GRU	63.3 %	63.3 %	64.09 %	62.80 %	39160
Proposed HHO optimized Bi-LSTM	95.64 %	99.64 %	91.63 %	95.47 %	2463

**Fig. 19:**
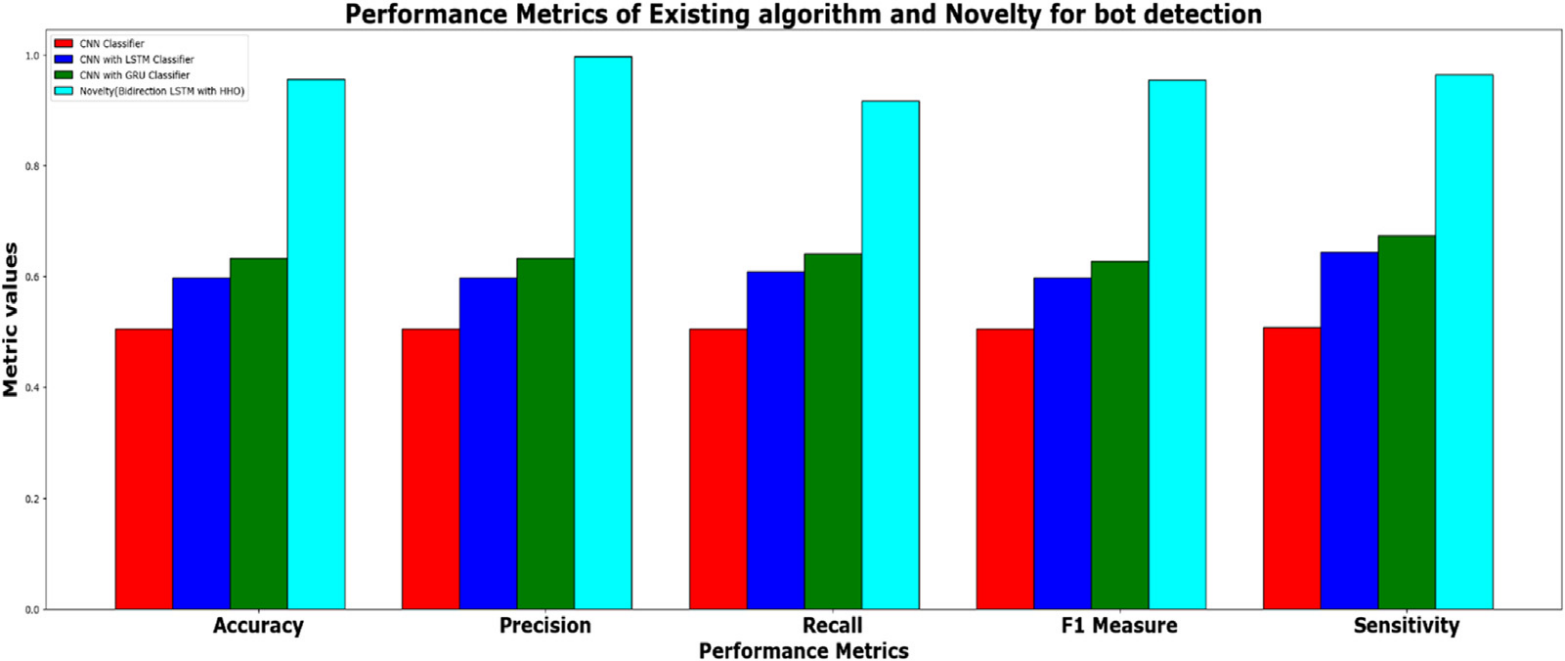
Performance evaluation of existing and proposed classifier for twitter bot dataset.

It can be inferred from Table 2 and Fig. 19 that the proposed HHO-BiLSTM achieved an optimal accuracy of 95.64 %, while the precision, recall, f1 score and support was found to be 99.64 %, 91.63 %, 95.47 % and 2463 respectively. The performance was validated by comparing the simulation results of the proposed approach with the existing Bi-LSTM model. Results state that the HHO-BiLSTM classifier performs better in terms of achieving superior accuracy.

### Simulation results for twitch bot dataset

The results of different performance metrics for the twitch bot dataset obtained from simulation are tabulated in Table 3.

**Table 3:** Performance metrics for the twitch chat bot dataset.

ML Models	Accuracy	Precision	Recall	f1 -score	Support
Naive Bayes	69.64 %	79.89 %	75.90 %	77.84 %	11232
SVM	76.86 %	77.05 %	86.81 %	76.00 %	11232
Random Forest	86.69 %	93.93 %	87.14 %	90.41 %	11232
Proposed Fuzzy Neuro with Hist Gradient Boosting Classifier	97.49 %	99.87 %	96.90 %	98.36 %	37438

**Fig. 20:**
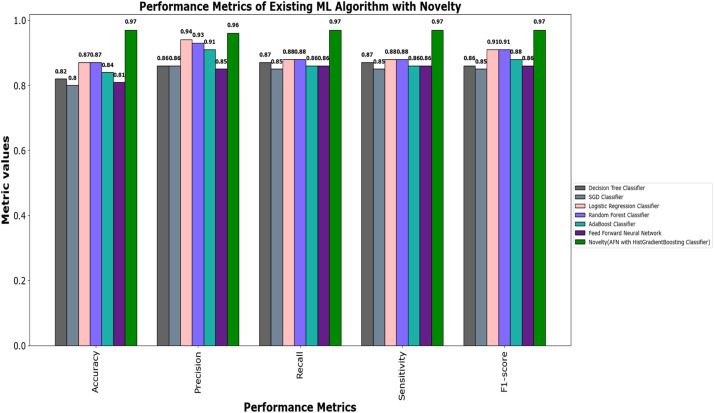
Performance evaluation of different ML classifiers for twicth bot dataset.

**Fig. 21:**
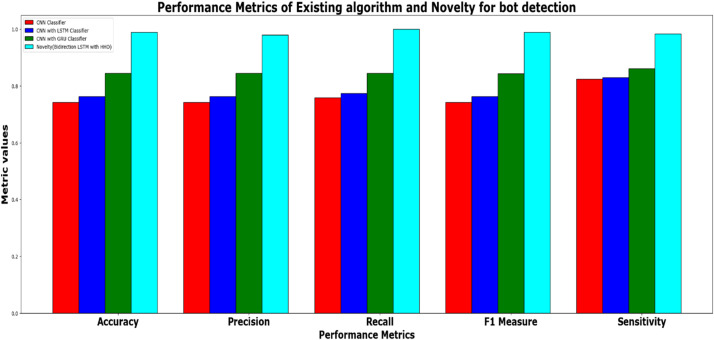
Performance evaluation of different ML classifiers for twitch bot dataset.

It can be inferred from the Table 3 and Figs. 20 and 21 that the Comparative analysis state that the HHO-BiLSTM classifier achieved an optimal accuracy of 97.49 %. The outcome of the HHO-BiLSTM classifier is compared with the outcome of the other existing classification models. Results validate the performance of the adaptive fuzzy with HGB classifier in terms of achieving a phenomenal accuracy with a very minimum false alarm rate (FAR) of 0.004113.

The HHO optimized Bi-LSTM is analyzed with respect to training and validation accuracy and loss.

**Fig. 22:**
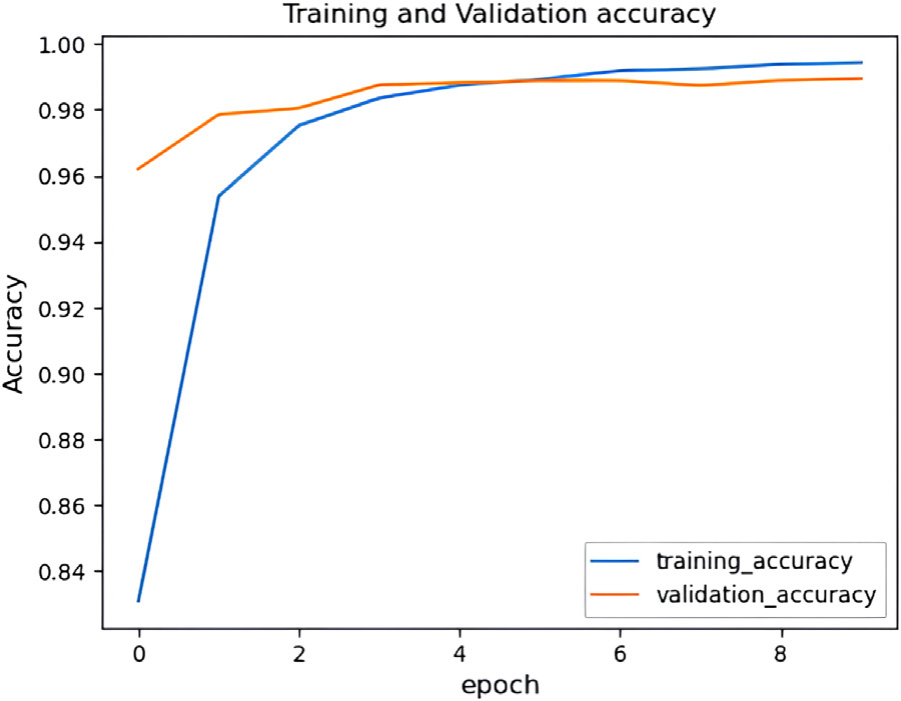
Training and validation accuracy of HHO-BiLSTM model for twitch bot dataset.

**Fig. 23:**
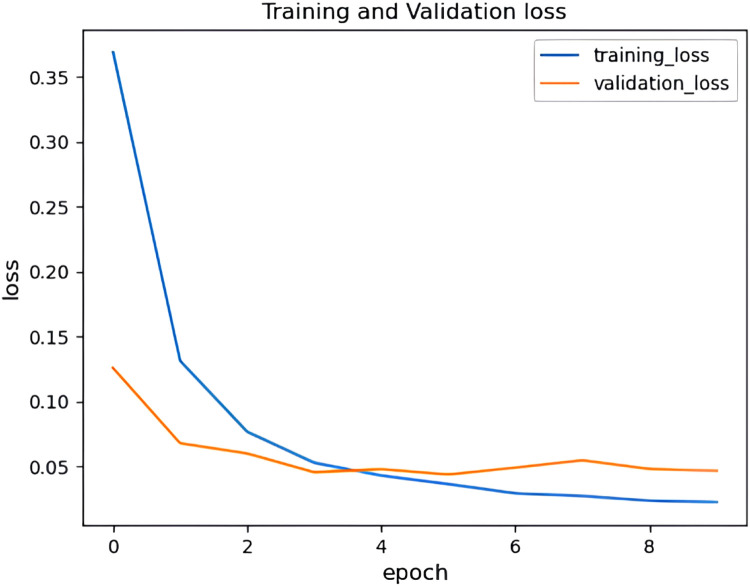
Training and validation loss of HHO-BiLSTM model for twitch bot dataset.

It can be observed from Figs. 22 and 23 that that the accuracy of the HHO-BiLSTM model is determined using both training and validation data. The graph shows that the validated accuracy is lower than the training accuracy. In addition to the performance evaluation, the performance of the proposed HHO optimized Bi-LSTM model is compared with existing Bi-LSTM model and the results are tabulated in Table 4**.**

**Table 4:** Performance metrics for the prediction model for twitter bot dataset.

ML Models	Accuracy	Precision	Recall	f1 -score	Support
Existing Bi-LSTM	61.31 %	57.0 %	34.32 %	47.01 %	7504
Proposed HHO optimized Bi-LSTM	98.98 %	98.03 %	100 %	99.00 %	26796

**Fig. 24:**
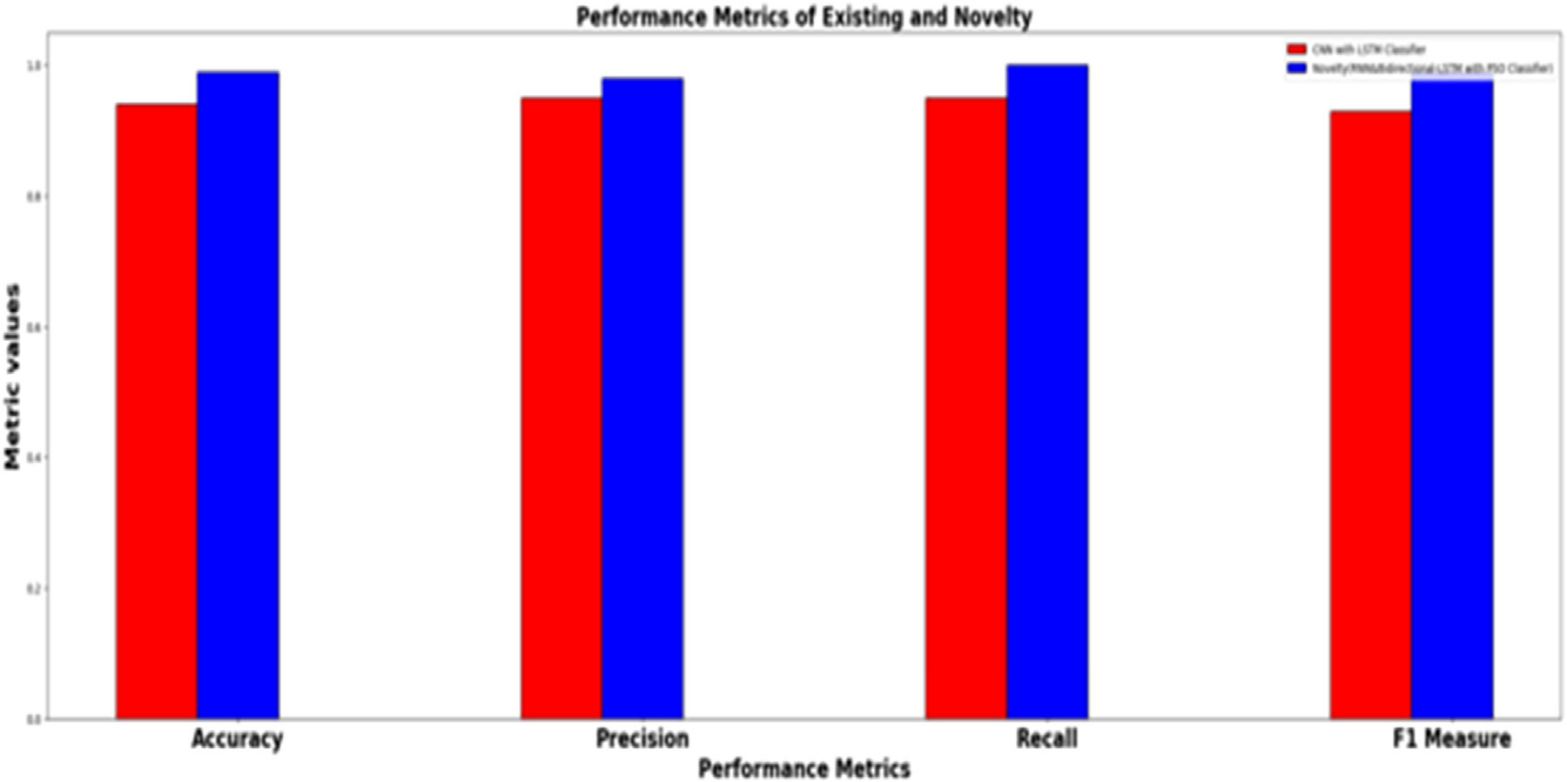
Performance evaluation of existing and proposed classifier for twitch bot dataset.

It can be inferred from Table 4 and Fig. 24 that the proposed HHO-BiLSTM achieved an optimal accuracy of 98.98 %. Results validate the performance of the HHO-BiLSTM model in terms of achieving superior accuracy. The fuzzy logic employed in this research enables the HHO-BiLSTM model to effectively classify the features which are not definitively bot-like or human-like. This is advantageous since the behavior of the social bots can be complex and not always binary. In addition, the integration of predictions generated by multiple decision trees has led to the formation of a robust and accurate model. Custom features are extracted from the data which are specific to the Twitter bot behavior and this contributed to the increased accuracy of the model. The adaptation of the fuzzy logic helped the model to adapt to changing bot behaviors over time, allowing the model to remain effective as bot tactics evolve. Furthermore, the combination of fuzzy logic and histograms can yield interpretable results, helping analysts and researchers understand why a particular account was flagged as a bot. By incorporating uncertainty handling and fine-tuning, the model achieved reduced false positives, which is crucial for maintaining trust and minimizing the impact on legitimate users.

## Conclusion

The study proposes a feature extraction based social bot detection model combined with an adaptive fuzzy neuro classifier and Hist. gradient boosting classification algorithm for detecting and predicting social bots. The proposed approach is a feature selection and hybrid learning based approach which combines two classifiers for automating the identification, and classification of social bots. With the adoption of a computationally efficient metaheuristic improved PSO algorithm for extracting relevant features, the proposed work is expected to identify and classify social bots and can achieve desired performance classification performance with minimum FAR. Simulation was conducted for both twitter bot dataset and twitch bot dataset. Results show that the HGB classifier exhibits excellent classification accuracy of 95.64 % for twitter bot dataset and 98.98 % for twitch bot dataset, compared to other existing ML models.

## Ethics statements

There were no animal experiments performed or animal subject used in the research work presented in this paper. Dataset obtained from twitter and twitch application are obtained from KAGGLE, an open-source platform. Hence, the KAGGLE's data redistribution policies were complied with.

## Additional information

The technological advancements in social bot detection have introduced different techniques for verifying the authenticity of the information shared. As discussed, in this paper, it is a challenging task to identify fake news from the abundant social media data. Majority of the ML models are related to classification and are not suitable for detecting fake data from real data [30] implemented a Bi-directional Long short-term memory (Bi-LSTM) and word embedding for detecting social bots in Twitter. The integration or word embedding with recurrent neural net- works improved the accuracy of detection and the results validate the efficacy of the proposed approach compared with existing detection systems when tested for cresci- 2017 dataset. A similar work is proposed in [31] which uses a word embedding technique for detecting social bots. The study implemented different ML algorithms such as logistic regression (LR) [32], Decision Tree (DT) [33], Support Vector Machine (SVM) [34], Random Forest (RF) [35], and Ensemble Learning (EL) [36] algorithm for determining the performance of bot detection models. These models were accompanied with different word embedding vectors such as BOW, TF-IDF, Doc2Vec, Bert, Word2Vec, and FastText. Results show that RF and DT exhibit superior performance compared to other algorithms. In addition to social bot detection, there is a great need to fact check the false (fake) news from the social media information.

A Random Forest and natural language processing (NLP) was employed for detecting fake news in) [37]. This study identified fake news by computing the similarity of the vectors that signify the content in the news that are used for words. This study identified fake news by computing counting the similarity of the vectors that signify the content in the news that are used for words. In addition, a RID matrix was also applied for calculating the similarity between the vectors. Despite the availability of various frameworks, it can be inferred from existing literary works that there is a lack of an effective technique which can fact check the available information aggregated from the social media sites and distinguish social bots from normal users hence the paper proposed a novel technique to aggregate the work.

## CRediT authorship contribution statement

**Monikka Reshmi Sethurajan:** Conceptualization, Methodology, Software, Data curation, Validation, Writing – original draft, Writing – review & editing. **Natarajan K．:** Visualization, Investigation, Supervision, Validation, Formal analysis, Project administration, Resources.

## Declaration of Competing Interest

The authors declare that they have no known competing financial interests or personal relationships that could have appeared to influence the work reported in this paper.

## Data Availability

Data will be made available on request.
